# Evaluating miR-122-5p as a biomarker for anti-tuberculosis drug-induced liver injury: diagnostic efficacy and correlation with liver function indicators

**DOI:** 10.7717/peerj.21911

**Published:** 2026-10-30

**Authors:** Guowei Ma, Chuang Yang, Yonggang Chen, Ji Luo, Xiang Zhu, Jie Chen

**Affiliations:** 1 Department of Pharmacy, The Third People’s Hospital of Kunming, Kunming, China; 2 College of Pharmacy, Dali University, Dali, China

**Keywords:** MicroRNA, Biomarker, Drug-induced liver injury, Diagnosis

## Abstract

**Background:**

Drug-induced liver injury (DILI) is characterized by hepatic dysfunction due to exogenous agents, potentially progressing to acute liver failure. MicroRNA-122-5p (miR-122-5p) constitutes about 70% of total liver microRNAs, offering high liver specificity and superior sensitivity for detecting injury compared to traditional transaminases, without interference from renal or muscle injury. This cross-sectional study aims to evaluate the diagnostic utility of miR-122-5p as a biomarker for anti-tuberculosis DILI.

**Methods:**

miR-122-5p expression was measured by real-time quantitative polymerase chain reaction (PCR). Descriptive statistics summarized sample characteristics. For normally distributed continuous variables with equal variances, data were presented as mean ± standard deviation, with comparisons among three groups using one-way analysis of variance (ANOVA); otherwise, data were expressed as median (interquartile range) with Kruskal–Wallis H test, followed by Mann–Whitney U pairwise comparisons. Categorical variables were analyzed by Chi-square test. Receiver operating characteristic (ROC) analysis assessed diagnostic efficacy (area under the curve (AUC), optimal cutoff, sensitivity, specificity). Correlations with alanine aminotransferase (ALT), aspartate aminotransferase (AST), and alkaline phosphatase (ALP) were evaluated by Spearman’s rank correlation, and scatter plots with fitted lines were generated using Python.

**Results:**

The DILI group showed significantly higher miR-122-5p expression (2.48 ± 0.81) than the Liver biochemical abnormality and Healthy control groups (*P* < 0.001). ROC analysis yielded an AUC of 0.829 (95% confidence interval (CI) [0.732–0.926]), with sensitivity 0.833 and specificity 0.760 for distinguishing DILI from non-DILI. miR-122-5p positively correlated with ALT (*ρ* = 0.626, *P* < 0.001) and AST (*ρ* = 0.522, *P* < 0.001), but not with ALP (*ρ* = 0.193, *P* = 0.114). No significant intergroup differences in age or gender were found (*P* > 0.05).

**Conclusions:**

Our research indicates that miR-122-5p has potential utility as a biomarker for anti-tuberculosis DILI, particularly in distinguishing it from healthy controls. However, these findings are exploratory, based on a small cohort with no independent validation, and its incremental value beyond conventional liver function tests remains to be determined.

## Introduction

Drug-induced liver injury (DILI) is a disorder characterized by hepatic dysfunction resulting from various exogenous agents, including pharmaceuticals, herbal remedies, natural substances, and minerals. In severe instances, DILI can progress to acute liver failure and potentially life-threatening conditions (Liu et al., 2020). Pathogenetically, DILI is typically classified into intrinsic, idiosyncratic, and indirect types, while acute DILI phenotypes are categorized into hepatocellular injury, cholestatic injury, and mixed injury based on the R value (Suk et al., 2012). Presently, DILI often manifests without distinct clinical symptoms in its early stages, and there is a notable lack of biomarkers with adequate sensitivity and specificity for diagnosis. This deficiency contributes to cases being missed or misdiagnosed and hampers accurate assessment of the actual incidence, thereby allowing DILI to progress insidiously to liver fibrosis, acute liver failure, and potentially result in patient mortality (Niu, Alvarez-Alvarez & Chen, 2025).

The incidence of DILI exhibits considerable regional variation, with the highest incidence reported in Asia (17.82 per 100,000 person-years, 95% CI [6.26–29.38]) and the lowest in North America (1.72 per 100,000 person-years, 95% CI [0.48–2.95]) (Li et al., 2023). In particular, the annual incidence of DILI within the general population of China is approximately 23.80 per 100,000, which is significantly higher than that observed in many other countries (Shen et al., 2019). Furthermore, the overall prognosis for Chinese patients with DILI is suboptimal, as evidenced by a cure rate of only 50.65% and a mortality rate of approximately 1.60% (Zhang et al., 2018). In China, acute DILI accounts for approximately 20% of hospitalizations related to acute liver failure (ALF), yet the precise incidence rate of DILI within the general population remains undetermined (Xiao et al., 2020). In contrast, in the United States, DILI is responsible for about 50% of acute liver failure cases, with a three-week survival rate of only 27.10% for non-transplanted patients suffering from DILI-related acute liver failure (Onakpoya, Heneghan & Aronson, 2019). This condition has emerged as a significant factor leading to the withdrawal or restricted use of drugs post-marketing (Reuben, Koch & Lee, 2010). Incidence rates reported in other countries include 2.70 per 100,000 in the United States (Reuben, Koch & Lee, 2010), 13.90 per 100,000 in France (Sgro et al., 2002), 19.10 per 100,000 in Iceland (Björnsson et al., 2013), and 12.00 per 100,000 in South Korea (Suk et al., 2012). The spectrum of drugs responsible for DILI exhibits regional variation. In Europe and the United States, nonsteroidal anti-inflammatory drugs, antimicrobials, and herbal/dietary supplements are the primary etiological agents (Chalasani et al., 2015). Conversely, in Asia and China, traditional Chinese medicine, anti-tuberculosis drugs, and antimicrobials are more prevalent, with traditional Chinese medicine/herbal/dietary supplements (26.81%) and anti-tuberculosis drugs (21.99%) identified as the most common individual causative agents in China. Hepatocellular injury is the predominant phenotype, accounting for 51.39% of cases (Devarbhavi et al., 2010). Several risk factors contribute to the development of DILI, including host genetic, immunologic, and metabolic factors, as well as drug-related and environmental influences (Chen et al., 2022).

Presently, the diagnosis of DILI is primarily a process of exclusion, utilizing the Roussel Uclaf Causality Assessment Method (RUCAM) scale in conjunction with standard liver function tests, such as alanine aminotransferase (ALT), aspartate aminotransferase (AST), alkaline phosphatase (ALP), and total bilirubin (TBIL) (Suk et al., 2012). However, traditional markers like alanine aminotransferase lack sufficient specificity to effectively differentiate liver damage of varying etiologies (Satoh et al., 2023). In recent years, novel biomarkers such as keratin 18, fatty acid-binding protein 1, glutamate dehydrogenase, and miR-122-5p have been investigated for their potential in diagnosing DILI (Church et al., 2019). Among these, miR-122-5p, which constitutes approximately 70% of total liver microRNAs (Lagos-Quintana et al., 2002), demonstrates exceptional liver specificity (Flanigan et al., 2014; Zhang et al., 2010) and exhibits superior sensitivity and specificity for detecting liver injury compared to traditional transaminases (Shi, Yang & Mendrick, 2013). Notably, the circulating levels of miR-122-5p remain unaffected by renal dysfunction or muscle injury (Vliegenthart et al., 2015; Thulin et al., 2014), and it possesses a predictive value of 70%–90% for subsequent liver injury in patients with normal or slightly elevated alanine aminotransferase levels (Antoine et al., 2013; Matthews et al., 2020). The upregulation of miR-122-5p is strongly associated with the downregulation of drug-metabolizing enzymes (DMEs), leading to increased drug accumulation in hepatocytes and hepatic tissues, thereby accelerating the pathogenesis of DILI (Gill et al., 2017; Chowdhary et al., 2017). Consequently, further validation of the diagnostic utility of miR-122-5p and the development of a more accurate, noninvasive diagnostic strategy for DILI are of significant clinical importance. In light of this context, the present study assessed the utility of miR-122-5p as a biomarker for the diagnosis of DILI.

## Materials & Methods

### Study design

This study prospectively collected plasma samples from inpatients at the Third People’s Hospital of Kunming City and from healthy controls recruited from the health check-up center during the same period, between August and November 2025. Participants were divided into three groups according to the following criteria. The DILI group was defined according to the *Guidelines for the Diagnosis and Treatment of DILI Induced by Anti-Tuberculosis Drugs (2024 Edition)*, with a RUCAM score ≥ 6 (*i.e.,* probable DILI). Patients with RUCAM scores of 3–5 (possible DILI) were excluded to ensure a robust reference standard. Because anti-tuberculosis drugs were administered as a combined regimen, causality was assessed for the regimen as a whole rather than for individual agents. The Liver biochemical abnormality group comprised patients with elevated serum ALT and/or AST levels (≥ 1.5 × upper limit of normal) but with a RUCAM score <3, and with confirmed alternative etiologies including viral hepatitis, alcoholic liver disease, or non-alcoholic fatty liver disease. The Healthy control group comprised individuals with no history of liver disease, normal liver function tests, and no acute or chronic illnesses, recruited from the health check-up center during the same period. A total of 68 participants were enrolled in this study, including 18 patients with DILI, 25 patients with liver biochemical abnormalities of non-DILI etiologies, and 25 healthy controls. The sample size was determined by the availability of eligible patients during the recruitment period. The study received approval from the Medical Ethics Committee of the Third People’s Hospital of Kunming City (Approval No. KSLL202401300017), and written informed consent was obtained from all participants.

### Inclusion criteria

The DILI group required participants to meet the following conditions: ① Adherence to the diagnostic criteria for DILI as specified in the *Guidelines for the Diagnosis and Treatment of Drug-Induced Liver Injury Induced by Anti-Tuberculosis Drugs (2024 Edition)*; ② assessment of the causal relationship between DILI cases and suspected drugs using the RUCAM scale, with inclusion limited to cases scoring ≥ 6 points.

### Exclusion criteria

① Duplicate enrollment; ② patients under 18 years of age; ③ presence of liver malignancies; ④ pregnant or lactating individuals.

### Sample collection and storage

Fasting venous blood samples (eight mL) were obtained from each participant through elbow venipuncture during the early morning hours within 48 h of DILI diagnosis and prior to any therapeutic intervention. Blood was collected into EDTA-coated tubes. Of the total volume, four mL was immediately allocated for the assessment of liver function indicators. The remaining whole blood was kept at 4 °C and centrifuged within 2 h of collection at 3,000 × g for 10 min at 4 °C to separate plasma. The plasma layer was then carefully transferred into RNase-free centrifuge tubes, aliquoted, and stored at −80 °C until further analysis. To minimise pre-analytical variability, samples underwent no more than one freeze-thaw cycle. Haemolysis was assessed by visual inspection of plasma for pink or red discolouration. No samples included in the final analysis showed evident haemolysis.

### RNA extraction and complementary DNA synthesis

Total RNA was isolated from plasma samples utilizing the BioTNT TRIzol^®^ RNA Extraction Reagent (Catalog No. A2010A0402), with rigorous quality control measures enforced throughout the experimental process. The optical density (OD) values of RNA at wavelengths of 260 nm and 280 nm (A260 and A280) were measured using a NanoDrop microvolume UV spectrophotometer. RNA samples exhibiting an A260/A280 ratio between 1.6 and 2.0 were deemed to possess acceptable purity and quality. The extracted total RNA samples were maintained in an ice bath to reach the freezing point. Subsequently, miRNA was reverse transcribed into complementary DNA (cDNA) employing stem-loop reverse transcription primers. The reverse transcription reaction system was prepared in accordance with the protocol provided by the BioTNT kit (Catalog No. A2030A001). The reverse transcription reaction was conducted at 42 °C for 60 min, followed by enzyme inactivation at 95 °C for 5 min using a Thermo qPCR instrument. The resulting cDNA samples were stored at −20 °C until further analysis of miRNA expression. The primer sequences used for miR-122-5p (MIMAT0000421) were as follows: forward primer, 5′-TGGAGTGTGACAATGGTGTTT-3′; reverse primer, 5′-CTCAACTGGTGTCGTGGAGT-3′. hsa-miR-16-5p was used as the internal reference gene, with forward primer 5′-GGGGTAGCAGCACGTAAA-3′and reverse primer 5′-TCAACTGGTGTCGTGGAG-3′. Quantitative real-time polymerase chain reaction (qPCR) was employed to evaluate the expression of miR-122-5p, using hsa-miR-16-5p as the reference gene. MiRNA expression levels were quantified using the BioTNT microRNA Realtime PCR PreMIX (Catalog no. A2030A002), following the manufacturer’s instructions. The stem-loop reverse transcription primer was used at a final concentration of 1 µM during the reverse transcription reaction. For qPCR, each 20 µL reaction mixture contained 10 µL of 2 × SYBR Green Master Mix, 0.5 µM of each primer, and 0.5 µL of cDNA template, with nuclease-free water added to the final volume. All reactions were performed in triplicate for each sample, and the standard deviation of Ct values was consistently below 0.18, indicating high reproducibility. The qPCR conditions were as follows: an initial denaturation step at 95 °C for 5 min, succeeded by 40 cycles consisting of denaturation at 95 °C for 5 s and annealing at 60 °C for 60 s, with fluorescence measurements taken at the conclusion of each cycle. Melting curve analysis was conducted from 72 °C to 95 °C, with fluorescence detection at each 0.3 °C increment for 10 s. The cycle threshold (Ct) values of the reference gene hsa-miR-16-5p were employed for normalization, and the relative expression level of miR-122-5p was determined using the 2^−^ΔΔCt method.

### Statistical analyses

All statistical analyses were executed using SPSS software (version 23.0). For continuous variables that adhered to a normal distribution and exhibited homogeneity of variance, data were presented as mean ± standard deviation (x¯ ± s), and comparisons among the three groups were performed using one-way analysis of variance (ANOVA). For data that did not conform to a normal distribution or homogeneity of variance, results were expressed as median and interquartile range (M(P25, P75)). Overall comparisons among the three groups were conducted using the Kruskal–Wallis H test. If a statistically significant difference was identified, pairwise comparisons were subsequently performed utilizing the Mann–Whitney U test. Categorical variables were reported as counts and percentages (n(%)), with intergroup comparisons conducted using the Chi-square (*χ*^2^) test, contingent upon the characteristics of the data. DILI was diagnosed according to the Guidelines for the *Diagnosis and Treatment of DILI Induced by Anti-Tuberculosis Drugs (2024 Edition)* with a RUCAM score ≥ 6, as described in the Methods section. Receiver operating characteristic (ROC) curve analysis was employed to assess the diagnostic efficacy of miR-122-5p in relation to DILI. The area under the curve (AUC) and its corresponding 95% confidence interval (CI) were calculated. The optimal cut-off value was established based on the maximum Youden index, from which sensitivity and specificity were derived. Correlations between miR-122-5p and liver function-related indicators, specifically alanine aminotransferase (ALT), aspartate aminotransferase (AST), and alkaline phosphatase (ALP), were evaluated using Spearman’s rank correlation analysis, including the computation of correlation coefficients and their statistical significance. Scatter plots with fitted trend lines were generated using Python. All statistical tests were two-sided, and a *P*-value of less than 0.05 was deemed statistically significant.

## Results

The baseline demographic characteristics of the three groups are summarized in Table 1. No significant differences were observed among the three groups regarding gender distribution (*χ*^2^ = 0.092, *P* = 0.955) or age (*F* = 1.509, *P* = 0.229), confirming the comparability of the groups. The RUCAM scores of the 18 DILI patients ranged from 6 to 8, with a median of 7 (data are presented as median (IQR): 6–7). Eighteen cases (100%) were classified as probable DILI. No cases were possible (3–5 points) or highly probable (>8 points). Among the 18 DILI patients, the majority of cases were induced by anti-tuberculosis drugs. The specific drugs implicated were isoniazid, rifampicin, ethambutol, and pyrazinamide. None of the patients required intensive care unit (ICU) admission or mechanical ventilation during the study period, and no DILI-related deaths occurred.

The miR-122-5p expression level in the DILI group was 2.48 ± 0.81, which was significantly elevated compared to both the Liver biochemical abnormality group and the Healthy control group (*P* < 0.001), indicating increased expression in patients with DILI. Post-hoc pairwise comparisons revealed that the miR-122-5p levels in the DILI group were significantly elevated compared to both the Liver biochemical abnormality group and the Healthy control group (*P* < 0.05). Furthermore, the miR-122-5p levels in the Liver biochemical abnormality group were significantly higher than those in the Healthy control group (*P* < 0.05) (Table 2).

In an exploratory ROC analysis, miR-122-5p showed potential diagnostic performance for DILI. When comparing the DILI group with the combined Liver biochemical abnormality and Healthy control groups, the AUC was 0.829 (95% Confidence Interval (CI) [0.732–0.926]), with a sensitivity of 0.833 and a specificity of 0.760 at the determined cut-off value. Stratified analyses indicated that the AUC for the DILI group *versus* the Liver biochemical abnormality group was 0.789 (95% CI [0.652–0.926]), with corresponding sensitivity and specificity values of 0.833 and 0.720, respectively. In comparison with the Healthy control group, the AUC was 0.869 (95% CI [0.760–0.978]), achieving a sensitivity of 0.944 and a specificity of 0.720 (Table 3).

The correlation analysis revealed that miR-122-5p showed a significant moderately strong positive correlation with ALT levels (*ρ* = 0.626, *P* < 0.001), indicating that elevated miR-122-5p is associated with increased ALT levels. Similarly, miR-122-5p demonstrated a significant moderate positive correlation with AST levels (*ρ* = 0.522, *P* < 0.001). Conversely, a weak positive correlation was identified between miR-122-5p and ALP (*ρ* = 0.193, *P* = 0.114), which was not statistically significant. Overall, miR-122-5p exhibited a more pronounced correlation with transaminases (ALT and AST), which are indicative of hepatocellular injury, whereas its association with ALP, an indicator related to cholestasis, was not significant (Fig. 1).

**Table 1:** Baseline demographic characteristics of the three study groups.

Characteristic	DILI group (*n* = 18)	Liver biochemical abnormality group (*n* = 25)	Healthy control group (*n* = 25)	*P* value
Age (years, mean ± SD)	50.44 ± 15.45	45.80 ± 15.95	41.76 ± 16.95	0.229
Age range (years)	24–76	19–77	19–78	–
Gender (male/female)	12/6	16/9	17/8	0.955

**Table 2:** Comparison of miR-122-5p levels among the three study groups.

Group	N	miR-122-5p	H value	*P* value
DILI group	18	2.48 ± 0.81	*H* = 20.971	<0.001
Liver biochemical abnormality group	25	1.77 (1.07, 2.11)^a^		
Healthy control group	25	1.38 (0.91, 1.68)^a^^b^		

**Notes.**

a *P* < 0.05 *vs.* DILI group.

b *P* < 0.05 *vs.* Liver biochemical abnormality group.

**Table 3:** Diagnostic performance of miR-122-5p for DILI.

	Youden index	Cut-off value	AUC	95% CI	Sensitivity	Specificity
DILI group *vs.* (Liver biochemical abnormality group + Healthy control group)	0.593	1.949	0.829	0.732∼0.926	0.833	0.76
DILI group *vs.* Liver biochemical abnormality group	0.553	1.932	0.789	0.652∼0.926	0.833	0.72
DILI group *vs.* Healthy control group	0.664	1.378	0.869	0.760∼0.978	0.944	0.72

**Figure 1:**
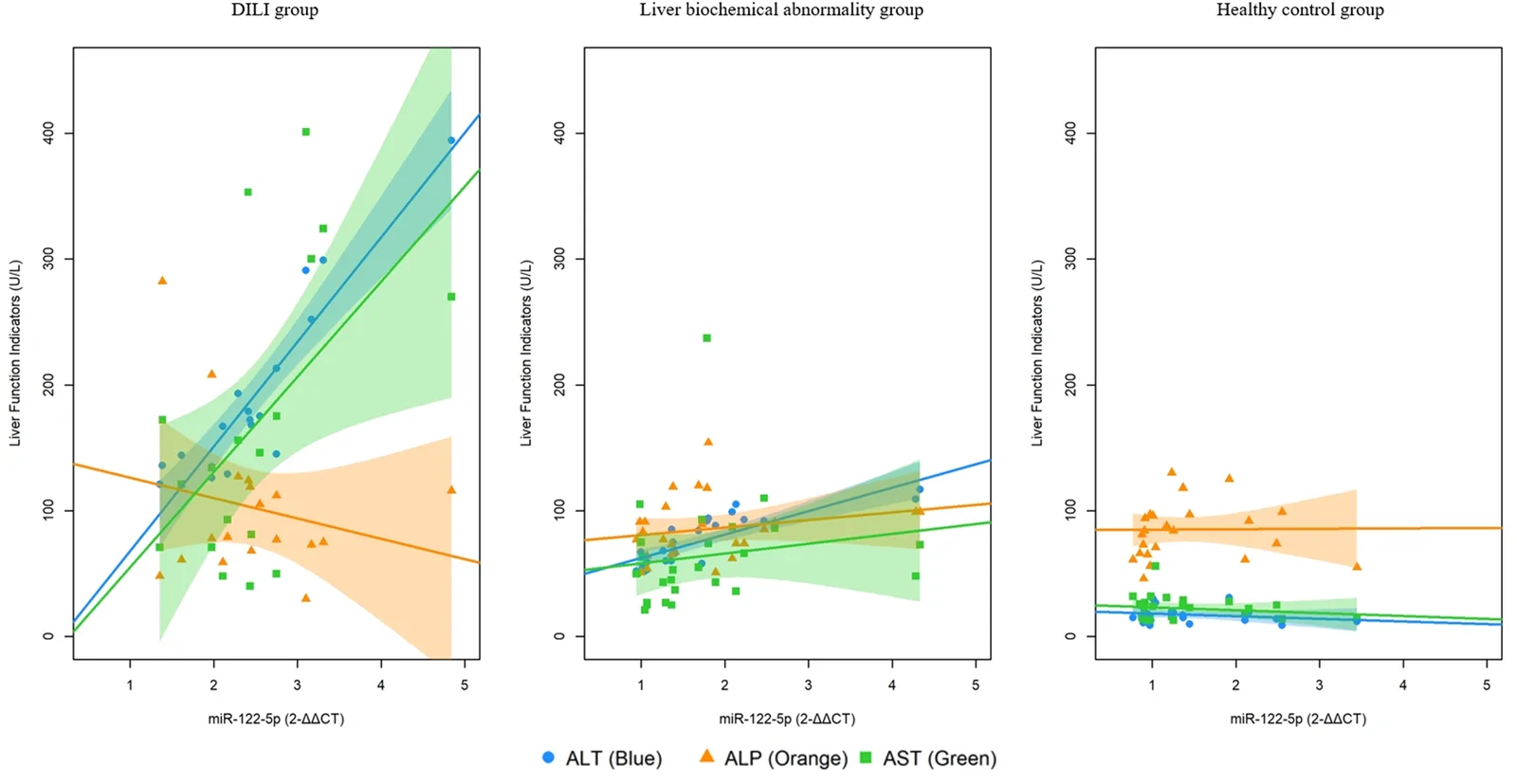
Correlation between miR-122-5p and liver function indicators in the DILI group, liver biochemical abnormality group, and healthy control group.

## Discussion

The markedly elevated levels of miR-122-5p observed in patients with DILI, particularly when compared to both healthy controls and individuals exhibiting non-DILI liver biochemical abnormalities, underscore its potential utility as a biomarker for differentiating DILI from non-DILI populations. This observation aligns with prior research indicating that miR-122-5p, a microRNA predominantly expressed in the liver, demonstrates a positive correlation with ALT and other indicators of hepatocyte damage (Zhang & Huang, 2021; Yang et al., 2021). The ROC analysis conducted in our study produced an AUC of 0.829, with a sensitivity of 0.833 and a specificity of 0.760, thereby reflecting potential discriminative ability. These findings further suggest that miR-122-5p may serve as a valuable adjunct to existing liver function tests, enhancing the identification and clinical risk assessment of DILI cases.

Beyond its diagnostic performance, miR-122-5p also offers several practical advantages over conventional markers. Although ALT is a traditional marker of liver injury, its specificity is limited due to potential elevation in certain non-hepatic pathological conditions, such as severe burns, muscle inflammation, and hypothyroidism (Shimoda & Kasai, 1980; Harr et al., 2008; Chang et al., 2004; Mitchell et al., 2008). In contrast, microRNAs are protected by extracellular vesicles and RNA-binding proteins, which confer high stability in circulation (Vickers et al., 2011; Ward et al., 2014) and a wide dynamic range, offering significant advantages for the prediction of drug-induced hepatotoxicity (Vliegenthart et al., 2014). As a liver-enriched microRNA, miR-122-5p is rapidly released into the bloodstream following hepatocellular damage and has been reported as a marker of hepatotoxicity in various experimental models and clinical studies (Lewis et al., 2011; Krauskopf et al., 2015; Vliegenthart et al., 2017). Its elevation is positively correlated with the severity of liver injury (Krauskopf et al., 2015).

In terms of correlations, we identified moderate positive correlations between miR-122-5p and both alanine ALT (*ρ* = 0.626, *P* < 0.001) and AST (*ρ* = 0.522, *P* < 0.001), which aligns with findings from earlier research (Lee et al., 2017; López-Riera et al., 2018; Zhang et al., 2025). This cross-study consistency indicates a stable co-variation pattern between miR-122-5p and transaminases, reinforcing its utility in evaluating liver injury. However, the correlation between miR-122-5p and ALP did not achieve statistical significance in our study (*ρ* = 0.193, *P* = 0.114), which partially diverges from some reports (Zhang et al., 2025). Potential explanations for this discrepancy include a limited sample size, which may result in insufficient statistical power, and the susceptibility of ALP to interference from factors such as bone metabolism and age. Therefore, the present findings do not eliminate the possibility of an association between these variables. Further research, incorporating larger sample sizes and stratification by distinct liver injury phenotypes, is necessary.

## Conclusions

In conclusion, miR-122-5p shows preliminary diagnostic potential as a circulating biomarker for anti-tuberculosis DILI, particularly in distinguishing it from healthy controls. However, these findings are exploratory, based on a small cohort with no independent validation, and its ability to differentiate anti-tuberculosis DILI from other causes of liver injury was more modest (AUC = 0.789). It also remains unclear whether miR-122-5p provides additional diagnostic value beyond conventional liver function tests. All 18 DILI cases in this cohort were induced by anti-tuberculosis drugs (isoniazid, rifampicin, ethambutol, and pyrazinamide), and no non-anti-tuberculosis drugs were identified as causative agents. Therefore, these findings should be interpreted as specific to anti-tuberculosis DILI and may not be generalizable to DILI caused by other agents. In addition, the single time-point design prevents assessment of the dynamic changes of miR-122-5p during the evolution and recovery of DILI. Future research should encompass more diverse DILI populations with independent validation to thoroughly assess its applicability.

##  Supplemental Information

10.7717/peerj.21911/supp-1 Supplemental Information 1 STROBE Checklist

10.7717/peerj.21911/supp-2 Supplemental Information 2 Statistics

10.7717/peerj.21911/supp-3 Supplemental Information 3 Data

10.7717/peerj.21911/supp-4 Supplemental Information 4 Translation codebook

10.7717/peerj.21911/supp-5 Supplemental Information 5 MIQE checklist

## Additional Information and Declarations

### Data Availability

The following information was supplied regarding data availability: The data is available in the Supplemental Files.
