# Supplementary material for: Evaluating miR-122-5p as a biomarker for anti-tuberculosis drug-induced liver injury: diagnostic efficacy and correlation with liver function indicators
Source: PeerJ. 2026 Oct 30;14:e21911. doi: 10.7717/peerj.21911 (PMC13726575; doi:10.7717/peerj.21911)
Supplement: Supplementary file 1 [file peerj-14-21911-s004.docx]

Translations for Supplementary_File.xlsx

Manuscript ID: 135663

Sheet 1:

判断 → Interpretation

特异单峰 → Specific single peak

数据可用 → Data available

数据不可用 → Data unavailable

2-ΔΔCT值 → 2-ΔΔCT value
